# Inconsistency of the “Sogo‐Shinryo” department in Japanese hospitals

**DOI:** 10.1002/jgf2.522

**Published:** 2022-01-31

**Authors:** Yuki Otsuka, Mikako Obika, Fumio Otsuka

**Affiliations:** ^1^ Department of General Medicine Okayama University Graduate School of Medicine, Dentistry and Pharmaceutical Sciences Okayama Japan

## Abstract

The inconsistency of Japanese names of “Sogo‐Shinryo” departments is also a greater problem in this country, and we are afraid that it may cause anxiety to patients, residents, and students. As one of the “Sogo‐Shinryo” departments in Japan, we want to provide them comprehensive learning environments regardless of the boundaries of academic cliques and societies, and, as a result, relieve them, foster general minds, and build their careers.

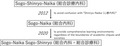

## CONFLICT OF INTEREST

The authors have stated explicitly that there are no conflicts of interest in connection with this article.

To the Editor,

We read the letter by Hideki Tsunoda and Kaku Kuroda, which considered and categorized the English translation of the “Sogo‐Shinryo” department of Japanese university hospitals.^1^ The viewpoint was greatly interesting, and it was a special report for objectifying the diversity of “Sogo‐Shinryo” or “General Medicine” in Japan.

They pointed out that there is spelling inconsistency in the English translation of “Sogo‐Shinryo.” We believe that this inconsistency is largely due to the inconsistency of the original Japanese name. Hence, the diversity of the Japanese name of each “Sogo‐Shinryo” department themselves should be more considered.

**FIGURE 1:**
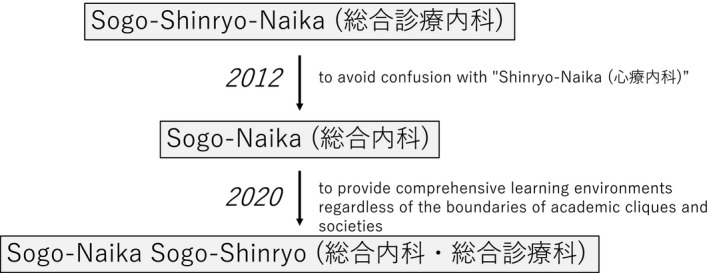
History of the Japanese name of the Department of General Medicine, Okayama University Hospital. The department has intentionally changed the Japanese name twice in this past decade

As the authors pointed out, the department's name represents the tasks and policies of each department. For example, our “Sogo‐Shinryo” department, which is consistently translated as “Department of General Medicine” in English and currently named “Sogo‐Naika Sogo‐Shinryo (総合内科・総合診療科)” department, was renamed from “Sogo‐Naika (総合内科)” in 2020. We renamed the department to a combination of “Sogo‐Shinryo” and “Sogo‐Naika” because we want to provide comprehensive learning environments for students and residents regardless of the boundaries of academic cliques and societies. Moreover, it was previously known as “Sogo‐Shinryo‐Naika (総合診療内科)” and changed in 2012 to avoid confusion with “Shinryo‐Naika (心療内科).” It almost corresponds to the psychosomatic department in English but is sometimes regarded by patients as the general medicine department due to the similarity in pronunciation (Figure 1).

It is natural that the names of departments are different between hospitals due to the different nuances they want to emphasize. In our department, organ‐specific specialists in internal medicine, hospitalists, and family physicians are mixed. We provide medical care as one department of a tertiary hospital, play roles as a hub center for regional community medicine, and educate students and residents. Probably, our function and vision or mission would not be entirely the same as other “Sogo‐Shinryo” department, and neither would the other departments.

However, there may be little clinical significance in separating them for those not involved.^2^ Moreover, the patients and the students and residents interested in becoming hospitalists or family physicians have a lot of anxiety in the current chaotic board certification system in Japan; both “Sogo‐Naika” and “Sogo‐Shinryo” specialists exist alongside. Thus, it would be most important to relieve them, foster general minds, and build their careers.
